# Three-dimensional in vivo analysis of water uptake and translocation in maize roots by fast neutron tomography

**DOI:** 10.1038/s41598-021-90062-4

**Published:** 2021-05-19

**Authors:** Christian Tötzke, Nikolay Kardjilov, André Hilger, Nicole Rudolph-Mohr, Ingo Manke, Sascha E. Oswald

**Affiliations:** 1 Institute of Environmental Science and Geography, University of Potsdam, Potsdam, Germany; 2 Institute of Applied Materials, Helmholtz Centre for Materials and Energy, Berlin, Germany

**Keywords:** Plant sciences, Environmental sciences, Optics and photonics

## Abstract

Root water uptake is an essential process for terrestrial plants that strongly affects the spatiotemporal distribution of water in vegetated soil. Fast neutron tomography is a recently established non-invasive imaging technique capable to capture the 3D architecture of root systems in situ and even allows for tracking of three-dimensional water flow in soil and roots. We present an in vivo analysis of local water uptake and transport by roots of soil-grown maize plants—for the first time measured in a three-dimensional time-resolved manner. Using deuterated water as tracer in infiltration experiments, we visualized soil imbibition, local root uptake, and tracked the transport of deuterated water throughout the fibrous root system for a day and night situation. This revealed significant differences in water transport between different root types. The primary root was the preferred water transport path in the 13-days-old plants while seminal roots of comparable size and length contributed little to plant water supply. The results underline the unique potential of fast neutron tomography to provide time-resolved 3D in vivo information on the water uptake and transport dynamics of plant root systems, thus contributing to a better understanding of the complex interactions of plant, soil and water.

## Introduction

A primary function of the root system is to extract water from the soil needed to balance the transpiration demand of the plant and to allow plant growth^1^. Root water uptake extensively contributes to terrestrial cycling of water back to the atmosphere and has a major impact on the spatiotemporal water content distribution in the upper layers of vegetated soils^2,3^. Due to intrinsic difficulties of observing belowground processes and assessing soil and root properties in situ*,* the basic understanding of root water uptake processes is still insufficient. While early work focussed on roots growing in hydro- or aeroponic culture^4–6^, later studies showed that root water uptake involves complex dynamic interactions between roots and the surrounding soil^7–9^. Recent advances in 3D neutron imaging have opened up new experimental approaches to study the hydraulic relations of roots and soil in vivo. Unlike proton or X-ray beams, a neutron beam interact only weakly with most mineral soil particles but strongly with hydrogenous substances^10–12^. For this reason, the presence of water in roots and in the soil matrix can be sensitively detect and localize^13^. Since the neutron beam also interacts differently with the different isotopes of hydrogen (essentially deuterium vs. hydrogen)^14,15^, water exchange processes between soil and roots can be followed using deuterated water (D_2_O) as contrast medium^16–19^. While 2D neutron radiography has already become a well-established tool to study dynamic water transfer phenomena in plants^18,20–23^ and soil^24,25^, partly including the use of deuterated water, the acquisition of 3D neutron tomograms has been too slow for resolving dynamic water transfer in root-soil systems, which restricted its application to the investigation of quasi-stationary systems^26^. With the development of high-speed neutron tomography, this limitation could be recently overcome^27^. Given sufficient neutron flux conditions at the imaging instrument, a full 3D image can be acquired in the range of a few seconds, which is sufficiently fast to visualize water infiltration in rooted soil columns^28^. The aim of the present study was to analyse water uptake and transport in the root system of young maize plants for the first time measured non-invasively in vivo in three dimensions.

## Results

Water uptake and transport through individual roots of three 13-days-old maize plants were monitored during infiltration experiments with deuterated water under day- and night-time conditions. The analysis of the plant sample “M14”selected as a good example is presented in detail while the results for the other two plants are summarized in the supplementary information (Fig. S1). The design of the experiment aims to quantify D_2_O infiltration and uptake by roots based on changes in neutron attenuation coefficients. In order not to stress the plants, which could inhibit transpiration and thus root water uptake, the soil water content must not be zero at the beginning of the experiment. Thus, injection of D_2_O results in some degree of mixing with the water already present in the soil, which is why pure D_2_O is not present in the soil solution. Thus, the D_2_O concentration in the soil solution determines the maximum D_2_O concentration in the root. However, the change in concentration over time can be used as a measure of root water uptake.

### Root system and water infiltration

**Figure 1:**
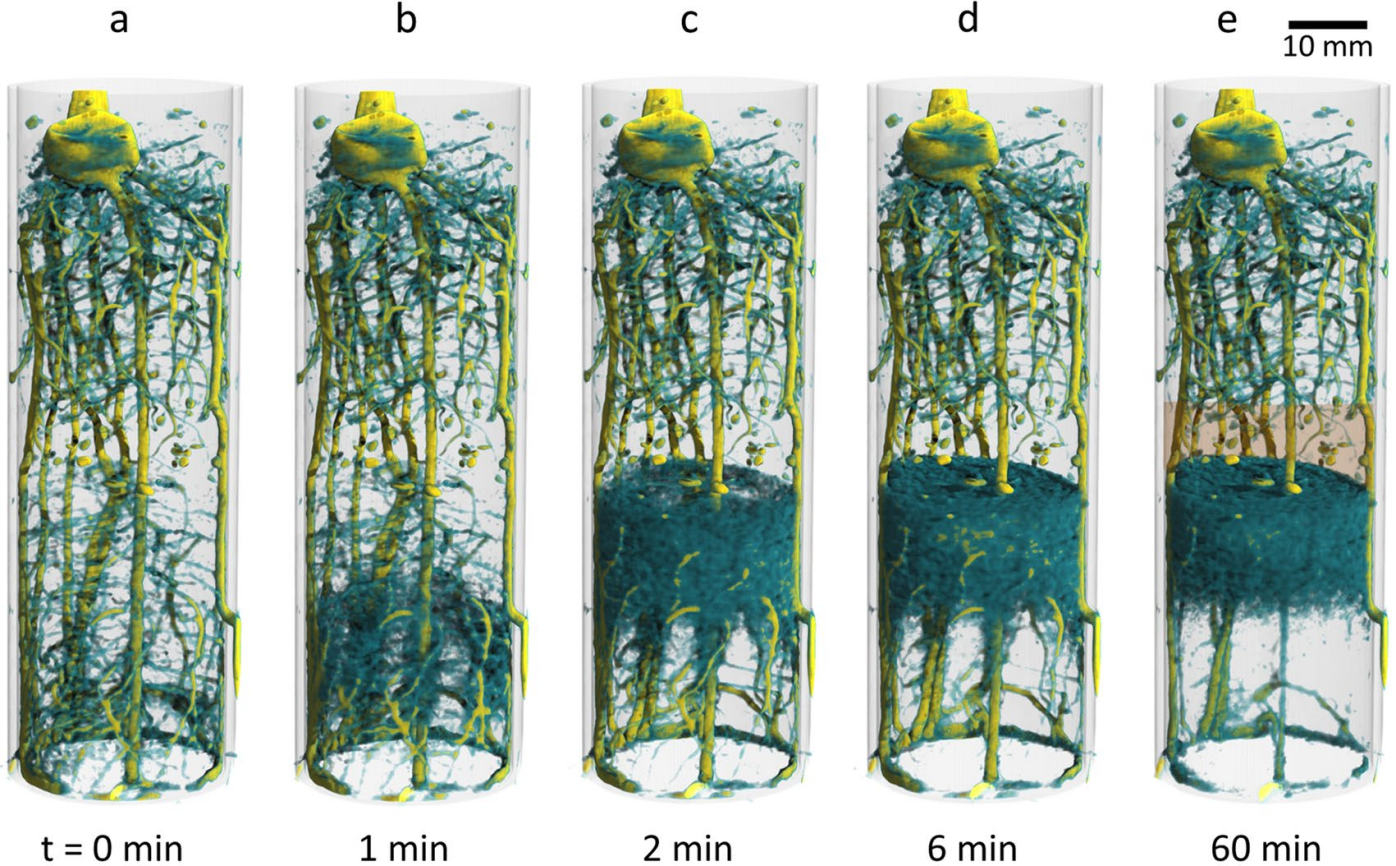
Time-resolved neutron tomography of a 13-days-old maize root system after infiltration of 4 ml deuterated water (D_2_O) through the bottom. (**a–d**) The 3D rendered the time series shows that D_2_O displaced the existing H_2_0 that built up to an upward moving water front and accumulated underneath the hydraulic barrier layer (indicted as brown-shaded area in (**e**). After 6 min, the dynamic part of soil water redistribution was completed. The volumetric water content of the lower soil compartment increased from θ_H2O_ = 0.1 to θ_liquid_ = 0.27 cm^3^/cm^3^ as result of the infiltration. Roots located in the bottom compartment appeared more transparent after 60 min indicating the flux of heavy water into the root system. The time resolution for each tomogram is 1 min.

Figure 1 illustrates the water movement in the soil column via capillary forces during infiltration of 4 ml of deuterated water from the bottom by a sequence of 3D-rendered tomographic images. The soil with a volumetric water content of 0.10 cm^3^/cm^3^ before infiltration provided excellent contrast for visualizing the seminal root system (Fig. 1a). It consisted of the primary root (coloured in red in Fig. 4a) and six seminal roots, all of which had numerous lateral roots. Primary and seminal roots had grown to the bottom of the container except for seminal root “6” (coloured in green in Fig. 4a). The latter exited the soil column via one of the lateral injection holes in the container wall. It continued growing outside for another centimetre trapped in between the container wall and the adhesive strip sealing the drilling hole. Light microscopy was used to obtain a detailed image of the internal structure of the water conduction system of young maize roots. Images of longitudinal and transversal cross sections of root segments excised from the hydraulic barrier layer are provided in the supplemental material (Fig. S2). The diameter of primary and seminal roots was in the range of 1 mm, while the stele was about 0.5 mm. The root stele containing the xylem vessels responsible for axial water transport was slightly wider in the primary compared to the seminal root.

**Figure 2:**
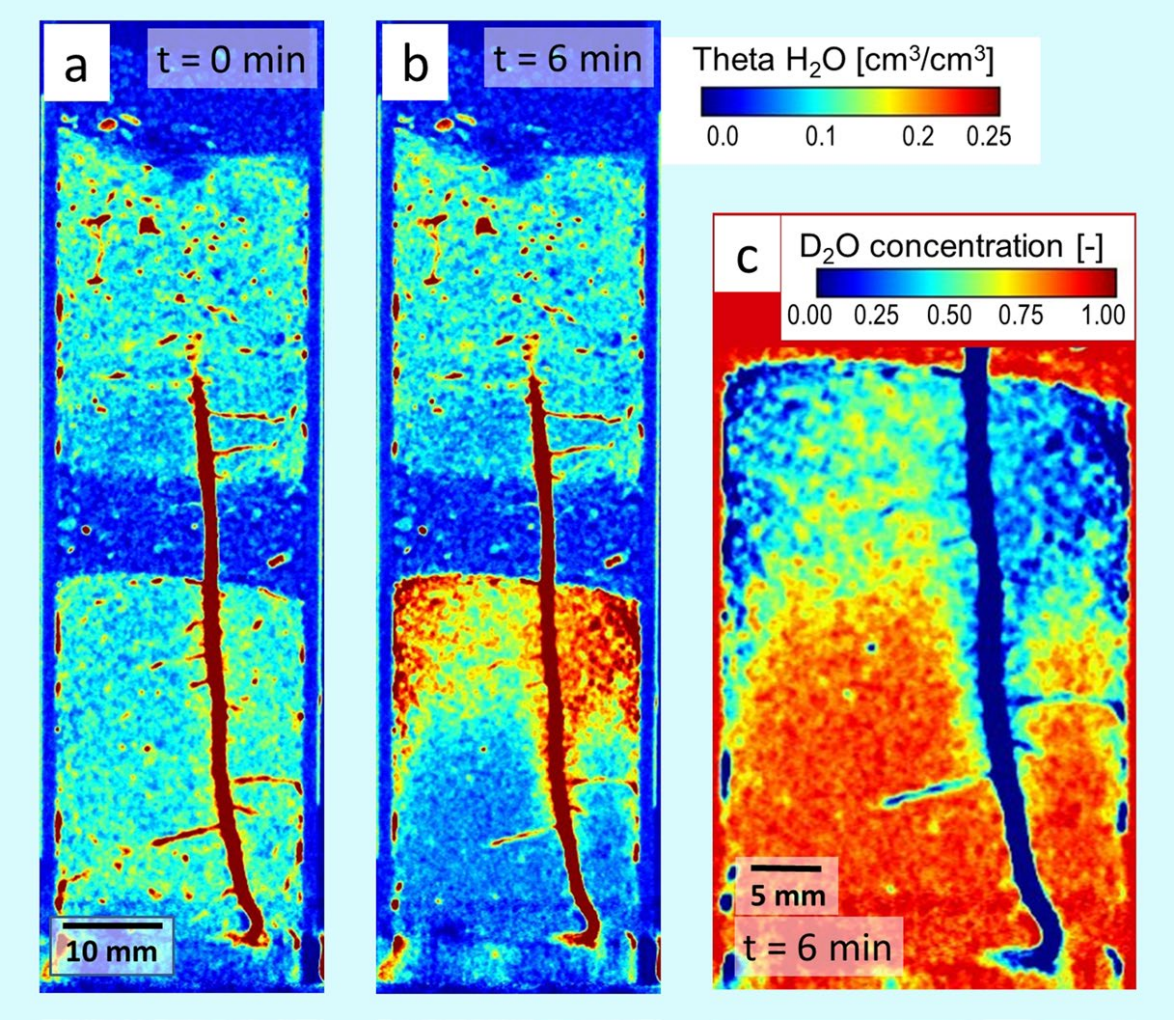
2D vertical cross section of the plant sample “M 14” taken at the position of the primary root during the experiment at daytime. (**a**) Distribution of the volumetric soil water (H_2_O) content before injection. The overall soil water content is θ_H2O_ = 0.10 cm^3^/cm^3^. (**b**) Distribution of the volumetric soil water (H_2_O) content after injection of 4 ml D_2_O. The total water (H_2_O + D_2_O) content in the lower soil compartment increased to θ_liquid_ = 0.27 cm^3^/cm^3^. Note that the colour scale was adapted to resolve the range of soil moisture and the calibration used does not apply to the roots. (**c**) Local D_2_O concentration of the soil water solution in the bottom compartment calculated under the assumption of a uniform total soil moisture of θ_liquid_ = 0.27 cm^3^/cm^3^.

Figure 1b shows the beginning infiltration of deuterated water. D_2_O invaded the pore matrix displacing soil water (H_2_O) that built up to an upward moving water front. In the tomographic image, this front appears as emerging turquois cloud, while infiltrating D_2_O is not visible here. Two minutes after infiltration, the H_2_O front approached the capillary barrier (Fig. 1c), a horizontal layer of coarse sand, where it stopped to form a sharp boundary (Fig. 1d). Six minutes after infiltration, the dynamic redistribution of water in the soil compartment slowed down and then stagnated. During the infiltration process, the soil moisture in the lower soil compartment increased from θ_H2O_ = 0.10 to θ_liquid_ = 0.27 cm^3^/cm^3^. Though mixing of the water components took place to some minor extend, D_2_O as water tracer had spread primarily within the lower 25 mm of the soil column while the displaced H_2_O had accumulated underneath the bottom of the barrier forming a layer of about 20 mm thickness. This general distribution persisted for the rest of the experiment (cf. Fig. 1c–e) indicating only slow mixing of deuterated with non-deuterated water in the pore matrix. The 10% higher density of deuterated water compared to H_2_O supports the stationary position of the tracer at the bottom of the soil column, which was advantageous for the intended observation of local D_2_O uptake by the roots. The spatial distribution of soil water (H_2_O) before and after infiltration is also presented quantitatively as 2D map for a vertical tomographic slice showing the primary root (Fig. 2a,b). Based on the H_2_O map (Fig. 2b), the distribution of D_2_O concentration in the soil water solution could be estimated under the simplifying assumption that the soil moisture was homogeneously distributed within in the lower soil compartment (Fig. 2c).

### Local root water uptake

**Figure 3:**
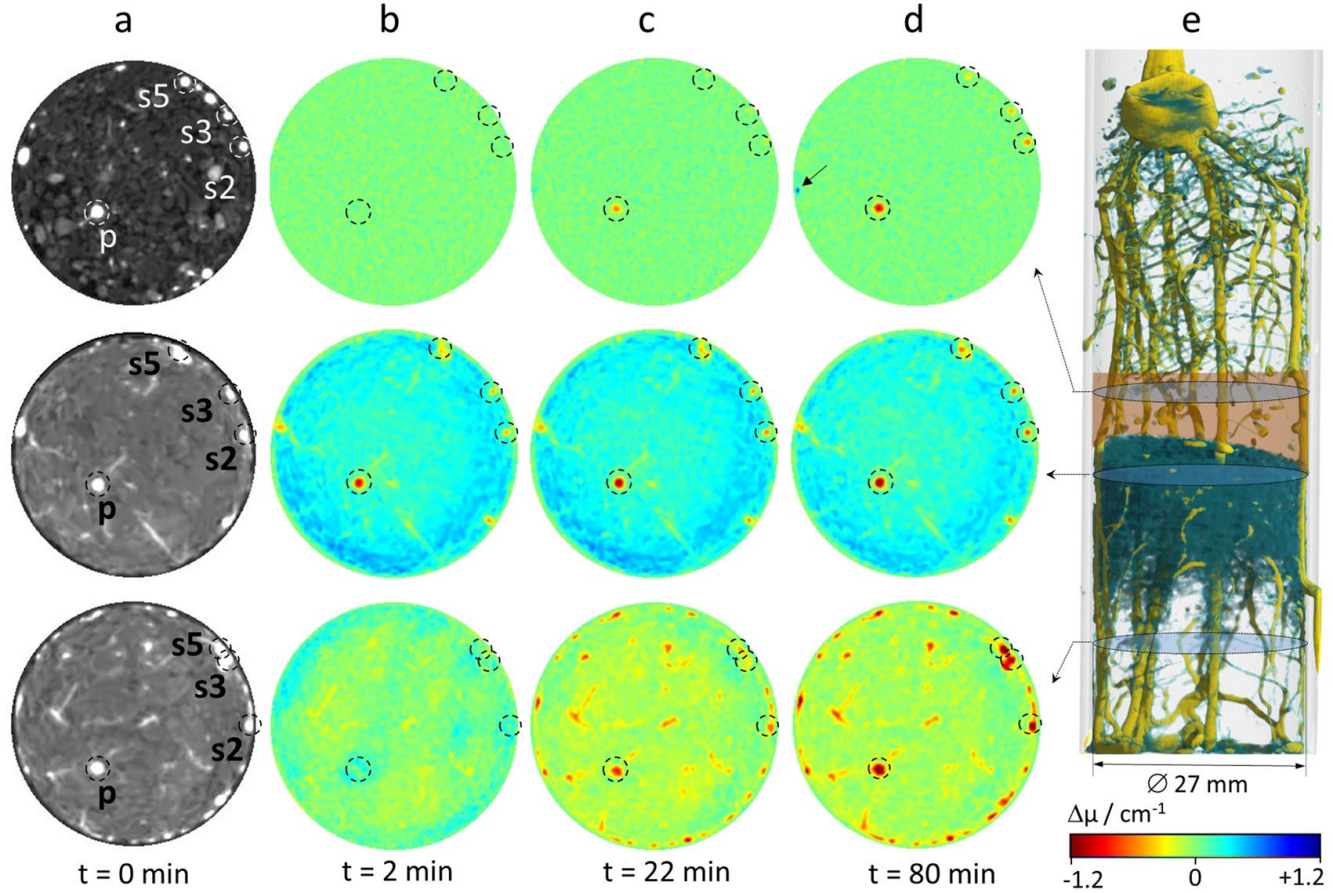
2D cross sectional views of a soil column with a 13-days-old maize root system displayed in regular grey scale (**a**) and as difference images with reference to t = 0 min (**b**-**d**). Position of cross sections in the root-soil column (3D-rendered at t = 6 min) is indicated in (**e**). Changes in neutron attenuation over time reveal the dynamic soil water redistribution and root uptake after infiltration of D_2_O at the bottom of the soil column. Primary (p) and three selected seminal roots (s2, s3 and s5) are marked with dashed circles. The colour bar displays the change in local neutron attenuation coefficient (Δµ).

All roots are clearly visible in the initial 3D rendered image (Fig. 1a) due to their high H_2_O content. However, 60 min after infiltration, roots located in the bottom compartment appeared more transparent or even disappeared visually indicating the flux of deuterated water into the root system (Fig. 1e). The evolution of the attenuation coefficients can be followed more precisely applying difference imaging. In Fig. 3, changes in local neutron attenuation coefficients associated with varying water content in soil and roots were calculated with respect to the initial image (taken before infiltration at t_Ref_ = 0 min). A selection of horizontal tomographic sections located at different heights of the soil column (marked in Fig. 3e) was chosen to represent soil zones with significantly different conditions for root uptake: (1) the D_2_O-rich zone in the bottom half of the compartment, (2) the H_2_O-rich zone closely beneath the barrier, and (3) the dry zone within the barrier. In the reference image (displayed in regular grey-scale in Fig. 3a), roots appear as brightest round-shaped structures since they are mainly composed of H_2_O, hence, strong neutron attenuators while dryer soil regions appear darker in this type of presentation. Positions of the primary (p) and three seminal roots (s2, s3, s5) are marked by dashed circles. The time series of difference images (Fig. 3b–d) displays progressing soil infiltration and subsequent root uptake at t = 2 min, 22 min and 80 min. Red colours indicate decreasing local attenuation, i.e. uptake of D_2_O by the root or displacement of soil water (H_2_O) by D_2_O. Blue colours point to increasing local attenuation as caused by increasing H_2_O content in the soil. Green colours refer to areas where the local attenuation coefficient did not change, which corresponds to stagnant H_2_O amount in the soil or no local D_2_O uptake by the root, respectively (cf. colour bar in Fig. 3).

The spreading of injected D_2_O and the related redistribution of H_2_O in the soil column determined the conditions for root water uptake during the experiment. The coarse sand matrix in the barrier provided constant dry conditions (θ < 0.01 cm^3^/cm^3^) as confirmed by stagnant attenuation coefficients (top row of Fig. 3b–d). This prevented local root uptake in this layer, thus changes in the attenuation coefficient of the root could be directly attributed to axial water transport. Note, the tiny water leak detected in the barrier layer (marked by an arrow in Fig. 3d, top row) did not allow water uptake as it was not in contact with the roots. Closely beneath the barrier layer (Fig. 3b–d, centre row), the attenuation over the entire soil cross-section shifted to significantly higher values, i.e. increasing H_2_O content, and remained on that level throughout the experiment. In this region, the roots were in direct contact with the accumulated water (H_2_O) displaced by the infiltration from below. At the base of the column, the D_2_O infiltration created a reservoir in the soil: the attenuation dropped over the cross section indicating displacement of existing H_2_O by D_2_O (Fig. 3b–d, bottom row).

As roots took up D_2_O from this reservoir, it successively replaced water in the root tissue triggering a drop of local attenuation in the round-marked root areas. D_2_O first entered the primary root (its round-marked cross section turned red in Fig. 3c, bottom row) and later also the seminal roots (Fig. 3d, bottom row). Further up in the H_2_O filled soil region, the decrease of attenuation in the roots was much smaller (Fig. 3b–d, centre row). Here, the signal is ambiguous as roots could take up H_2_O, but also receive D_2_O by axial transport taken up further below. The only slight decrease of attenuation indicates that both processes contributed and H_2_O taken up locally was diluting the D_2_O in the stream from below. However, the high soil water content in this zone entailed low neutron transmission rates promoting pronounced artefacts (i.e. neutron scattering artefacts and enhanced beam hardening) that impair the quantification of attenuation coefficients and complicate the interpretation of root transport within this soil zone. To avoid these uncertainties, the evaluation of axial water transport was focused on the barrier layer (top row of Fig. 3) where the arrival of D_2_O was first detected in the primary root 22 min after infiltration, while no change could yet be detected in the seminal roots. After 80 min, attenuation coefficient of the primary root had strongly decreased, while the decrease was less pronounced in the seminal roots (Fig. 3d, top row). Compared to the D_2_O-rich soil zone below, the decrease in attenuation is substantially smaller for all roots (cf. Fig. 3d bottom row vs. top row).

### Root water transport differences between a day and night situation

**Figure 4:**
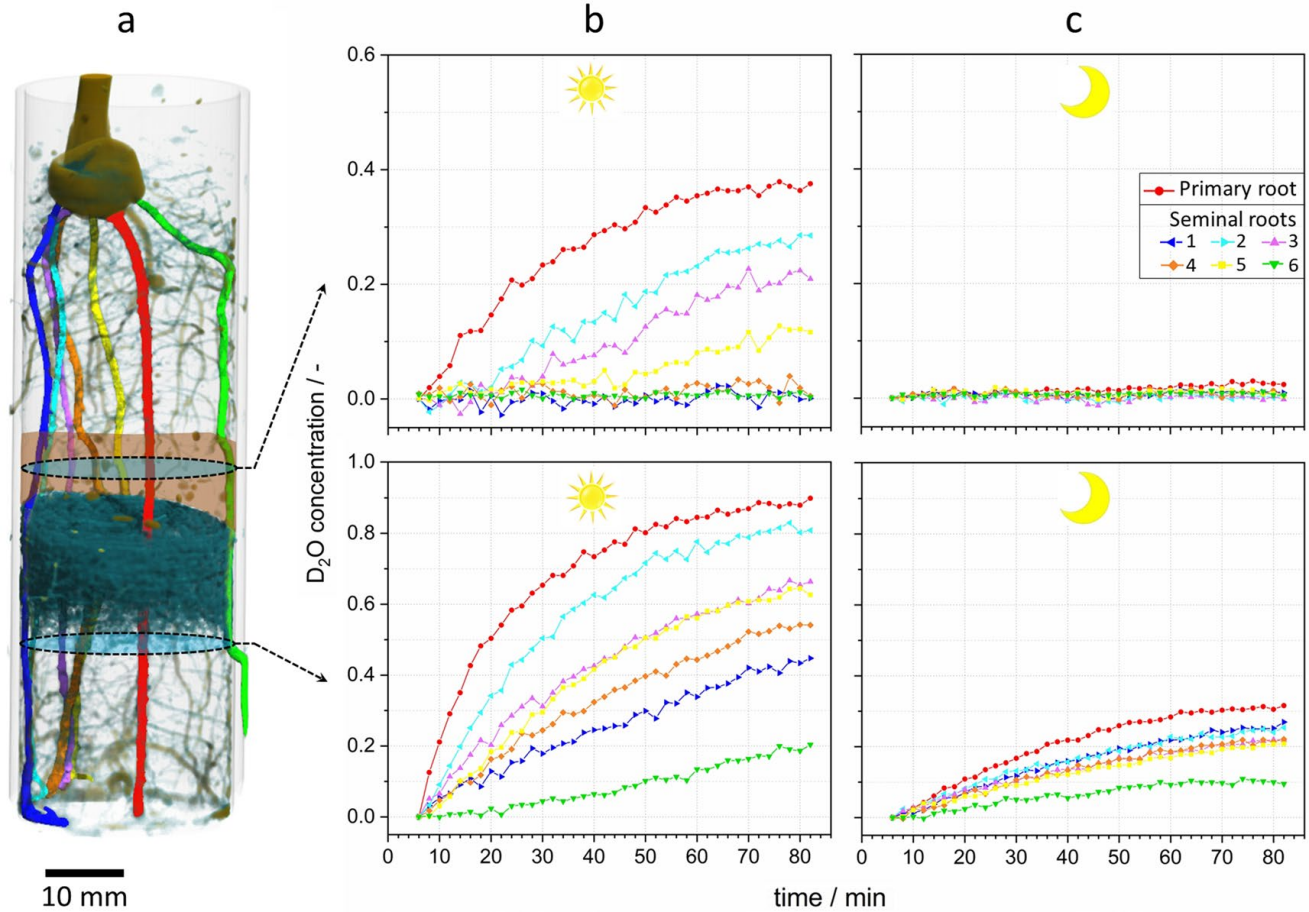
Development of D_2_O concentration (fraction of D_2_O in the total water content of the root) in individual root segments at two different heights of the root system reflecting root water uptake and transport during a daytime and night-time experiment. (**a**) 3D rendering of the maize root system. The turquois cloudy structure represents the soil water agglomeration underneath the barrier (brown-shaded area). The positions of evaluated root cross sections are indicated by blue-shaded ellipses. Primary and seminal roots are highlighted following the colour scheme of the plots. (**b**) and (**c**) Plots of D_2_O concentration for individual roots measured during the day vs. night experiment.

The analysis of root water uptake and transport was elaborated further by calculating the D_2_O concentrations (C_D2O_) for root sections located in the bottom region and the barrier layer. The course of the primary and six seminal roots was tracked throughout the soil column in order to correctly assign the D_2_O concentration at the selected positions (Fig. 4a). During the daytime experiment, the transpiration rate of the maize plant was 0.60 g/h compared to 0.09 g/h during the night-time experiment. The tomogram captured at t = 6 min was chosen as reference for difference imaging in order to minimized the influence of soil water fluctuations on the calculation of D_2_O concentrations in the root. At this point, the dynamic redistribution of the soil water had just been completed. The steepest increase of D_2_O concentration to a value of C_D2O_ = 0.4 was observed for the primary root during the first 15 min of experiment (red graph in Fig. 4b, bottom) indicating the highest individual root uptake rate. During the same period, the D_2_O concentration in the seminal roots increased at significantly smaller rates while seminal root “s6” remained almost constant. This root had only limited exposure to D_2_O as it grew to the outside of the container through a borehole located just below the considered position (green coloured root in Fig. 4a). As discussed above the absence of root water uptake in the hydraulic barrier allows for assigning changes in D_2_O concentration to axial root water transport. The plot of D_2_O concentrations in Fig. 4b, top confirms that the distinct rates of roots uptake observed in the bottom compartment corresponded to different rates of axial transport in individual root sections observed above in the dry barrier layer. There is a striking difference in the evolution of D_2_O concentration between primary and seminal roots. Within the first 15 min, the concentration increased to C_D2O_ = 0.12 in the primary root but remained nearly zero for all seminal roots. After 80 min, D_2_O concentration in the tissue of the primary root increased to 0.38 while only ranging between 0.28 and 0.12 in the seminal roots “s2”, “s3” and “s5” or even remaining at zero level for roots “s1”, “s4” and “s6”. This suggests the primary root supplied the major part of the water to the young plant while seminal roots only contributed moderately or seemed to be not active. In order to understand why some of the seminal roots did not transport the D_2_O they apparently took up in the bottom region; it is useful to recall the two possible mechanisms for D_2_O uptake: i) transpiration-driven convective water transport and ii) radial diffusion of D_2_O driven by the gradient of tracer concentration between soil and root. To illustrate that the root system takes up deuterated water also in the near absence of a transpiration stream, the experiment was repeated at night without illumination. The consistent result is displayed in Fig. 4c: At the bottom, were the soil water is rich in D_2_O, the tracer entered the roots via the radial diffusion path as proven by the similar increase of D_2_O concentration in all roots. The only notable exception is seminal root “s6”, the one with limited access to the reservoir of deuterated water. In contrast, D_2_O concentrations of all root sections in the barrier layer remained at zero level, proving that D_2_O in root segments below did not ascend in the root xylem because of the transpiration stream being almost absent.

### 3D tracking of water translocation in the entire root system up to the stem

**Figure 5:**
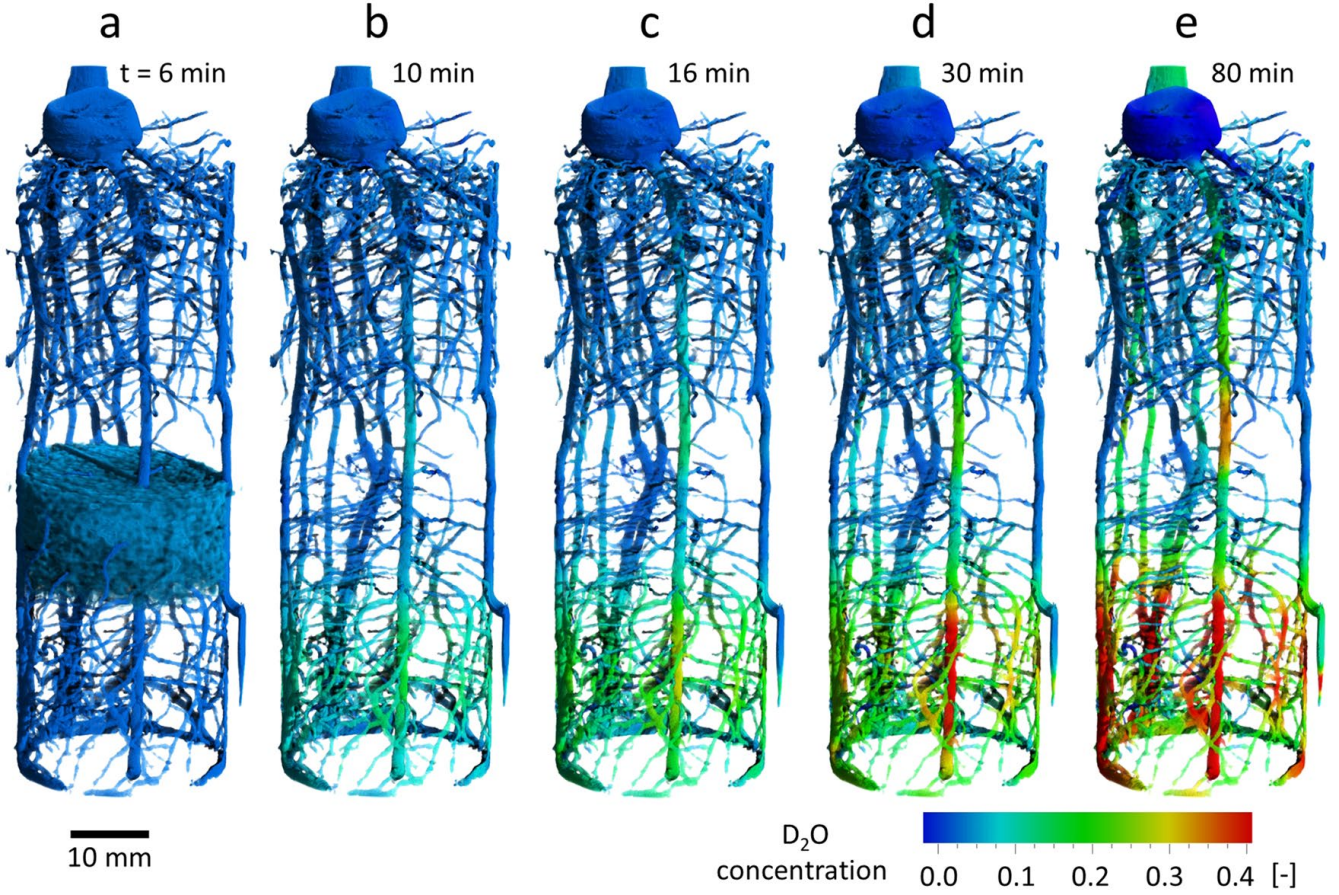
Tomographic time series of the segmented maize root system showing the evolution of local D_2_O concentration in the root system reflecting water uptake and axial transport by individual roots. (**a**) Reference image taken six minutes after D_2_O infiltration, when the soil water distribution had become stagnant. The soil water (H_2_O) accumulation is displayed as blue cloudy structure. The soil region underneath is interfused with deuterated water (transparent). (**b**–**e**) Development of D_2_O concentration in the tissue of the individual roots. The time resolution for each tomogram is 2 min. A video of this sequence is provided in the supplementary information.

Up to this point, data analysis was based on the choice of representative tomographic slices (2D). Fast neutron tomography, however, allows for visualizing dynamic root water uptake and axial transport in three dimensions. Segmentation of the root system and subsequent calculation of difference images revealed the temporal change in D_2_O concentration for each point in the 3D root system (Fig. 5, Video S1). Figure 5a depicts the reference image taken six minutes after D_2_O infiltration including the H_2_O accumulation beneath the barrier layer. Figure 5b illustrates the start of root uptake of D_2_O at the bottom of the soil column noticeable by green-shift of roots. At t = 16 min after infiltration, the conducting tissue of the primary root had already transported deuterated water above the barrier layer while the ascent of D_2_O in the seminal roots “S2”, “S3” and “S5” was significantly slower or not even detectable for the remaining seminal roots (see Fig. 5c–e and consult Fig. 4a to recall root labelling). At t = 80 min the stem base shifted green indicating that D_2_O had arrived even at the above ground part of the maize plant. The evaluation of the 3D root system confirmed that the primary root is the major contributor to the water supply of the 13-days-old maize plant. The results of the other two maize plants studied (“M16” and “M17”) are in agreement with this observation. Water ascent predominantly occurred in the primary root (Fig. S1b and S1c, top row in the supplementary information) while it was very small or even negligible in the seminal roots. The transpiration rate of the plant “M16” during the daytime experiment was 0.43 g/h and 0.63 g/h for plant “M17”. Note when comparing plant “M16” with “M17” that the lower D_2_O concentration in the roots was related to the different initial soil water content. While the infiltration of 4 ml D_2_O increased the volumetric water content in the lower soil compartment of sample "M17" from θ_H2O_ = 0.06 to θ_H2O+D2O_ = 0.23, it increased from θ_H2O_ = 0.19 to θ_H2O+D2O_ = 0.37 for sample “M16”. Hence, the soil of “M16” was considerably wetter, i.e. the tracer became much more diluted during infiltration.

## Discussion

The presented tracer experiment demonstrates that 3D water uptake and subsequent transport in fibrous root systems of young maize can be analysed in vivo in a time-resolved manner using fast 3D neutron tomography. During D_2_O infiltration, soil water was dynamically redistributed in the bottom of the plant container. After 6 min, the conditions for water uptake had stabilized. The roots took up D_2_O from the reservoir at the bottom and transported it upward with the transpiration stream. However, as the roots could also take up water when traversing the H_2_O-rich soil layer above, deuterated water ascending in the root xylem became diluted to some degree before it arrived in the barrier layer. At daytime, root uptake of D_2_O from the soil involved two simultaneous mechanisms: convective transport into the root via the transpiration stream and radial diffusion driven by the differences in D_2_O concentration between soil pore space and root tissue. As demonstrated in the night experiment, root uptake by radial diffusion also takes place in the absence of the transpiration stream. Therefore, this contribution to the increase of D_2_O concentration should not be considered as direct indicator for advective water uptake but merely as superposed diffusion process of the contrast medium. Evaluating individual root sections in the barrier layer, the predominant importance of the primary root for the water supply of the 13 days old maize plants became evident. Although the plants had already formed numerous seminal roots of comparable diameter, these roots transported significantly less or no water, which may point either to yet low axial root conductance limiting the transport capacity of the conducting tissue or to limited soil water availability at the root surface. Because highly concentrated D_2_O was distributed throughout the soil of the lower half of the bottom compartment (Fig. 2c), limited water availability could be excluded for the seminal roots. This was also confirmed by the similar nocturnal diffusion of D_2_O into the roots (Fig. 4c). It is therefore reasonable to attribute the significant higher water transport observed in the primary root to its higher water transport capacity. In general, the conductivity of roots varies over time with progressing level of xylem maturation^29,30^. While the architecture of the seminal root system was already well-developed 13 days after planting, the xylem maturation seemed not yet completed (c.f. the light microscopic images of the 10-days old roots presented in Fig. S2). The root system appeared at a stage of development where axial water flow was largely restricted to the lower conductivity provided by the protoxylem and to narrower vessels of the early metaxylem, since the much more efficient wide lumens of the metaxylem were not yet conductive^31,32^. Compared to the seminal roots xylem maturation in the primary root had already been able to advance further as this root is the first one grown by the seedling. Furthermore, it had already developed a larger number of lateral roots which contribute to a larger absorbing surface and are thought to play an important role in water uptake of young maize plants^23^. The higher degree of maturity of the vascular tissue and the increased absorption surface made the primary root a preferred water supply path for the young maize plants. As the root system continues to develop, the importance of the primary root for water supply may gradually become smaller as the conductivity of the seminal roots increases^31^. Later, with the emergence of nodal roots, the plant will gain further effective water uptake routes^33^.

The accuracy of the presented results is determined by the spatiotemporal resolution of the tomographic method. Temporal stability in the measurement of the attenuation coefficients was high (relative error of < 2%, as shown in Fig. S4). However, beam hardening and scattering artefacts intrinsically related to the physical interaction of sample with non-monochromatic neutron beam represent another limitation. If beam hardening and scattering artefacts only play a minor role, which is the case for relatively dry samples, the water distribution can be quantified with an estimated error of 5%. However, when it comes to dynamic redistributions of larger quantities of soil water, scattering artefacts may complicate the quantitative evaluation of local attenuation in the root tissue considerably. To avoid significant errors caused by a fluctuating scattering pattern on the neutron detector, the analysis of root water uptake by difference imaging considered a reference image taken 6 min after infiltration when the water distribution in the surrounding soil had already stabilized. Further improvements in the quantification of dynamic 3D water transfer processes are expected, once procedures for dynamic scattering corrections become established. Performing high-speed neutron tomography at even more powerful neutron facilities compared to BER II, e.g. at Institute Laue-Langevin in Grenoble, France^34^ or the future neutron source at the European Spallation Source in Lund, Sweden^35^, holds further potential. The larger neutron flux provides suitable conditions for monochromatic measurements avoiding beam hardening effects, which often cause significant problems in quantitative measurements. The stronger signal could also be exploited to refine the spatial resolution for studies on local root water uptake or for acceleration of the image acquisition process. The latter could be particularly useful for infiltration experiments designed to study water pathways in the soil layer surrounding the roots, termed rhizosphere. Time resolved neutron tomography can provide key information for current modelling approaches, which attempt to integrate three-dimensional root architecture, root growth and function explicitly in order to reliably predict the water and nutrient uptake of plants^3,36,37^.

## Materials and methods

This work reports on non-invasive imaging experiments with young, potted maize plants. Plants were cultivated at the University of Potsdam and transported to the nearby cold neutron imaging instrument CONRAD II operated by the Helmholtz Centre for Materials and Energy Berlin (HZB).

### Plant growing

For reasons of transmissivity, cylindrical plant containers are well suited for 3D neutron experiments. Since moist soil strongly attenuates neutrons, the maximum diameter of the plant containers is limited to about 30 mm. Individual maize plants (*Zea mays *L.* subsp. mays*) were grown in quartz glass columns of 27 mm inner diameter and 100 mm height filled with sandy soil collected at the catchment “Hühnerwasser” near Cottbus, Germany (cf.^7,17^). At half height, a 1 cm thick horizontal layer of coarse sand divided the soil column into two compartments, blocking capillary water transport without hindering roots to grow across (see e.g.^27^). Seeds were germinated for 48 h, and then planted at 1 cm soil depth. After sprouting, a 1 cm thick layer of gravel was added on top in order to minimize evaporation from the soil surface. Plants were grown in a plant growth chamber providing 14 h of light per day with an intensity of 400 μmol/m^2^ s. The day temperature of 24 °C was reduced to 19 °C at night, while the relative air humidity was kept at 60%. Plants were watered every second day from top and bottom to reach a soil water content of 0.25 cm^3^/cm^3^. Eleven days after planting the maize samples were transferred to the imaging facility and stored in a plant growth chamber with identical growing conditions. Two days before the imaging experiment, watering was stopped. During tomographic acquisition at daytime plants were illuminated from above by a plant-growing lamp with a light intensity of 400 μmol/m^2^ s to sustain transpiration. Applying the mass balance of the plant sample before and after tomography allowed the estimation of the transpiration rate during the experiments:

1 $$ \dot{T} \approx M_{sample, before} + M_{D2O} - M_{sample, after} $$

where M_sample_ is the weight of the plant sample before and after the measurement and M_D2O_ the weight of infiltrated deuterated water. Evaporation from the soil surface was neglected, which appeared reasonable as the deuterated water was injected into the lower soil compartment. Furthermore, a layer of gravel covered the surface of soil to minimize evaporation. During daytime experiments, the transpiration rate ranged from 0.44 to 0.63 g/h during night, it dropped to 0.09 g/h.

### Imaging experiments

Tomographic experiments were performed at the neutron imaging instrument CONRAD II, which was provided with a high and stable flux of cold neutrons by the research reactor BER II ^38^. The detector system consisting of a scintillator screen, a mirror and a 2D detector (sCMOS, Andor Neo) was located 5 m downstream of the neutron pinhole of the imaging instrument. The pinhole aperture was 3 cm resulting in a L/D ratio of 167. A complete tomographic scan over 360° included 600 radiographic projections with single exposure time of t = 0.2 s amounting to a total acquisition time of 2 min. The pixel size of the camera was adjusted to 55 µm. Pixel binning (2 × 2) was applied to improve signal-to-noise ratio, which led to an effective pixel size of 110 µm and a physical spatial resolution of approx. 220 µm. The field of view was adapted to 55 × 140 mm^2^ (width × height). The signal-to-noise ratio for radiographic projections and their corresponding flatfield images was 7.0 and 28.0, respectively. The contrast between roots and soil was about 18% in the radiographic projections and about 43% in the tomographic slices^27^. Given the high neutron contrast between hydrogen and its isotope deuterium, deuterated water (D_2_O) is an ideal tracer for tracking the replacement and transfer of water (respective thermal neutron attenuation coefficients are: µ(D_2_O) = 0.68 cm^−1^ vs.^.^µ(H_2_O) = 3.53 cm^-1^) ^39^. As the structure of D_2_O only differs by an additional neutron in the hydrogen nuclei, it is chemically very similar to H_2_O and has a high plant tolerance, which is necessary to ensure undisturbed root water uptake and subsequent axial transport. At the start of the experiment, the plant container was placed closely in front of the scintillator, rotated at constant speed of 0.5 rpm and irrigated from below with 4 ml of D_2_O by a syringe pump (Fresenius Pilot C) with an infiltration rate of 1.67 ml/min. Though faster tomogram acquisition is possible, the scanning time was set to 2 min per 360° tomogram, which allowed both resolving the dynamics of root water uptake and capturing the fibrous root system in appropriate detail. This scanning speed met the higher signal-to-noise ratio requirements needed for root difference imaging. The first six minutes after infiltration were resolved with 1 min per tomogram by reducing the angular range from 360° to 180° in order to capture the faster water infiltration of soil. The most relevant imaging parameters are summarized in Table S1 (see supplementary information).

In order to resolve the conducting system of the maize roots on a cellular level, transversal and longitudinal cross sections of primary and seminal roots were prepared and imaged by light microscopy at the Institute of Biochemistry and Biology at the University Potsdam. The plants were grown under the same conditions as the plants studied in the neutron imaging experiment but already harvested after 10 days for logistical reasons.

### Imaging processing

This study required the evaluation of time-lapse sequences of 3D neutron images with each tomogram containing a series transmission radiographs, i.e. angular projections of the plant sample. Inhomogeneity of the beam profile and the camera background were corrected using the beam profile captured without the specimen (flat field images) and images captured with the beam shutter closed (dark field images). Any variations in intensity of the incident neutron beam were normalized, using a sample-free region of the detector as reference. This guaranteed high temporal stability in the measurement of the attenuation coefficients, as shown in Fig. S4. Using a filtered back projection algorithm in batch routines programmed in IDL (Harris Spatial Resolution, Broomfield/USA, https://www.l3harrisgeospatial.com/Software-Technology/IDL) the projection images were reconstructed into a virtual 3D volume of the sample. The steps of the reconstruction process are illustrated in the supplementary information: Fig. S3a shows a raw image of the angular projection, Fig. S3b the same normalized angular projection, and Fig. S3c,d tomographic slices of the sample as a result of the reconstruction process. The reconstructed 3D image is composed of voxels each of which representing the average local attenuation coefficient $\overline{\mu }$ of the materials present at the time t in the sub-volume at the position of the voxel (x, y, z)

2 $$ \overline{\mu }\left( {x,y,z,t} \right) = \mathop \sum \limits_{i = 1}^{n} \cdot F_{i} \left( t \right)\mu_{i} $$

where F_i_ is the fraction of sub-volume occupied by the material i. For voxels containing soil, this reads

3 $$ \overline{\mu }_{soil} \left( t \right) = \left( {1 - {\Phi }} \right) \cdot \mu_{soil, dry} + \vartheta_{H2O} \left( t \right) \cdot \mu_{H2O} + \vartheta_{D2O} \left( t \right) \cdot \mu_{D2O} $$

where Φ is the porosity and µ_soil,dry_ is the attenuation coefficient of the dry soil. The 2D distribution of soil water (H_2_O) was derived from calibration measurements using the same soil with defined soil water contents.

The attenuation coefficient of a voxel containing roots is given by

4 $$ \overline{\mu }_{root} \left( t \right) = F_{H2O} \left( t \right) \cdot \mu_{H2O} + F_{D2O} \left( t \right) \cdot \mu_{D2O} + F \cdot \mu_{root tissue, dry} $$

The exchange of H_2_O by D_2_O during root uptake was tracked via difference imaging evaluating the local change in the attenuation of the roots. In order to minimize the influence of artefacts resulting from dynamic redistribution of water in the surrounding soil we used the image captured only 6 min after D_2_O infiltration when soil water had distribution became stagnant.

5 $$ \Delta \mu_{root} \left( t \right) = \mu_{root} \left( t \right) - \mu_{root} \left( {t = 6\;{\text{min}}} \right) $$

Assuming the root tissue at the reference time still being free of D_2_O (root uptake and transport is much slower compared to the water infiltration of soil) and a constant volumetric liquid content of the root tissue, the concentration of D_2_O in the root tissue can be estimated as

6 $$ C_{D2O} = \frac{{\Delta \mu_{root} \left( t \right)}}{{\mu_{D2O} - \mu_{root} \left( {t = 6\;{\text{min}}} \right)}} $$

C_D2O_ represents the fraction of D_2_O in the total water content of the root tissue and was averaged over the horizontal cross section of individual roots. The three-dimensional representation of water uptake and transport required a virtual extraction of the roots system from the soil. The segmentation algorithm programmed in IDL for this purpose was based on 3D hysteresis thresholding. Rendering of the 3D images presented in Fig.s 1, 3e, 4a, 5, Fig S1a + d, and Video S1 was performed using the software VGSTUDIO Max Version 3.1 (Volume Graphics, Heidelberg/Germany, www.volumegraphics.com). The 2D images presented in Figs. 2, S3, S4, 5, and Fig S1a + d were generated using ImageJ (https://imagej.net).

### Ethics declaration

All plants study has been conducted in accordance to the general guidelines and the authors comply with the IUCN Policy Statement on Research Involving Species at Risk of Extinction and the Convention on the Trade in Endangered Species of Wild Fauna and Flora.

## Supplementary Information

Supplementary Figures and Table.

Supplementary Video 1.

## Data Availability

The datasets generated during the current study are available from the corresponding author on reasonable request.
