# Supplementary material for: Three-dimensional in vivo analysis of water uptake and translocation in maize roots by fast neutron tomography
Source: Sci Rep. 2021 May 19;11:10578. doi: 10.1038/s41598-021-90062-4 (PMC8134433; doi:10.1038/s41598-021-90062-4)
Supplement: Supplementary file 1 — Supplementary Figures and Table. [file 41598_2021_90062_MOESM1_ESM.pdf]

Supplementary material to

"Three-dimensional *in vivo* analysis of water uptake and translocation in maize roots by fast neutron tomography"

Christian Tötzke[1*], Nikolay Kardjilov[2], André Hilger[2], Nicole Rudolph-Mohr[1], Ingo Manke[2], Sascha E. Oswald[1]

[1]Institute of Earth and Environmental Science. University of Potsdam, Potsdam, Germany
[2]Institute of Applied Materials, Helmholtz Centre for Materials and Energy, Berlin, Germany

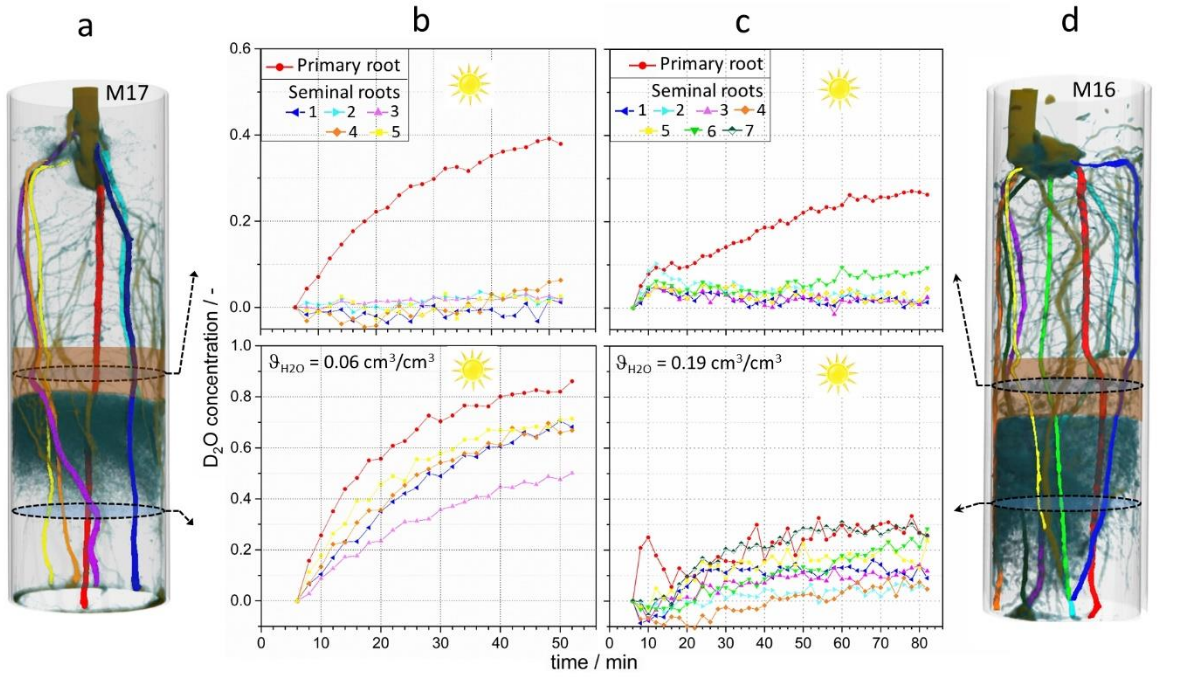

Figure S1. Development of D_2O concentration reflecting the water uptake and transport of individual roots of two young maize plants in a daytime experiment. (**a**+**d**): 3D rendering of sample "M17" and "M16". The turquois cloudy structure represents the soil water accumulation underneath the barrier (brown shaded area). Positions of evaluated root cross sections are indicated. Primary and seminal roots are highlighted using the colour scheme of the plots. (**b** + **c**) plots of D_2O concentration for individual root segments of sample "M17" and "M16". Note that the tracer diluted more during infiltration of the lower compartment of "M16", as it was significantly wetter than in the lower compartment of "M17".

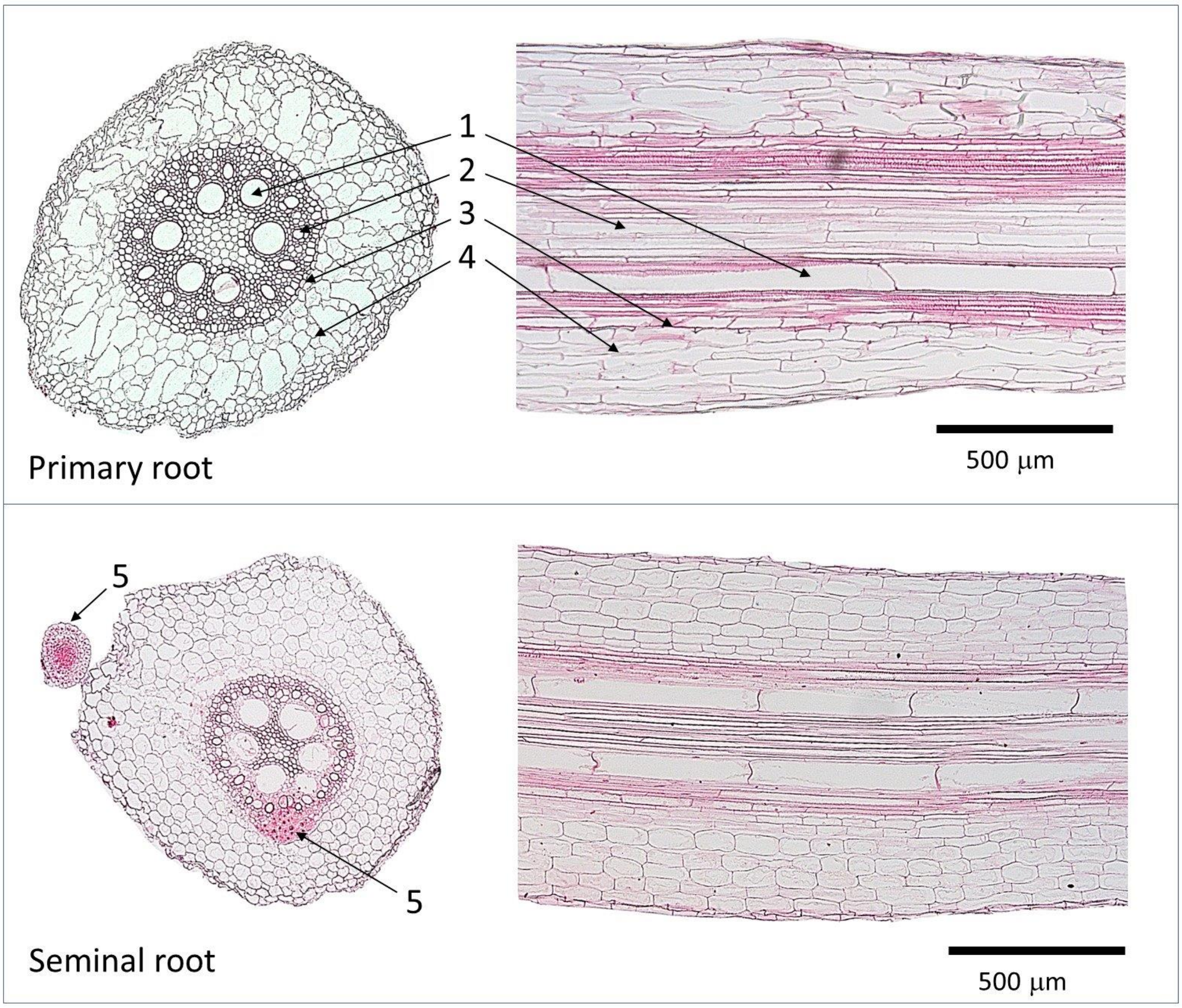

Figure S2. Light microscopic images of a primary and seminal root excised from a 10-days-old maize root system grown under same conditions as the plants studied in the neutron experiments. Transversal (left column) and longitudinal (right column) cross sections were prepared by microtome sectioning from root segments located within the barrier layer of the container. Numbered arrows mark essential structural features: (1) metaxylem conduits, (2) xylem conduits, (3) endodermis, (4) cortex, (5) emerging lateral roots.

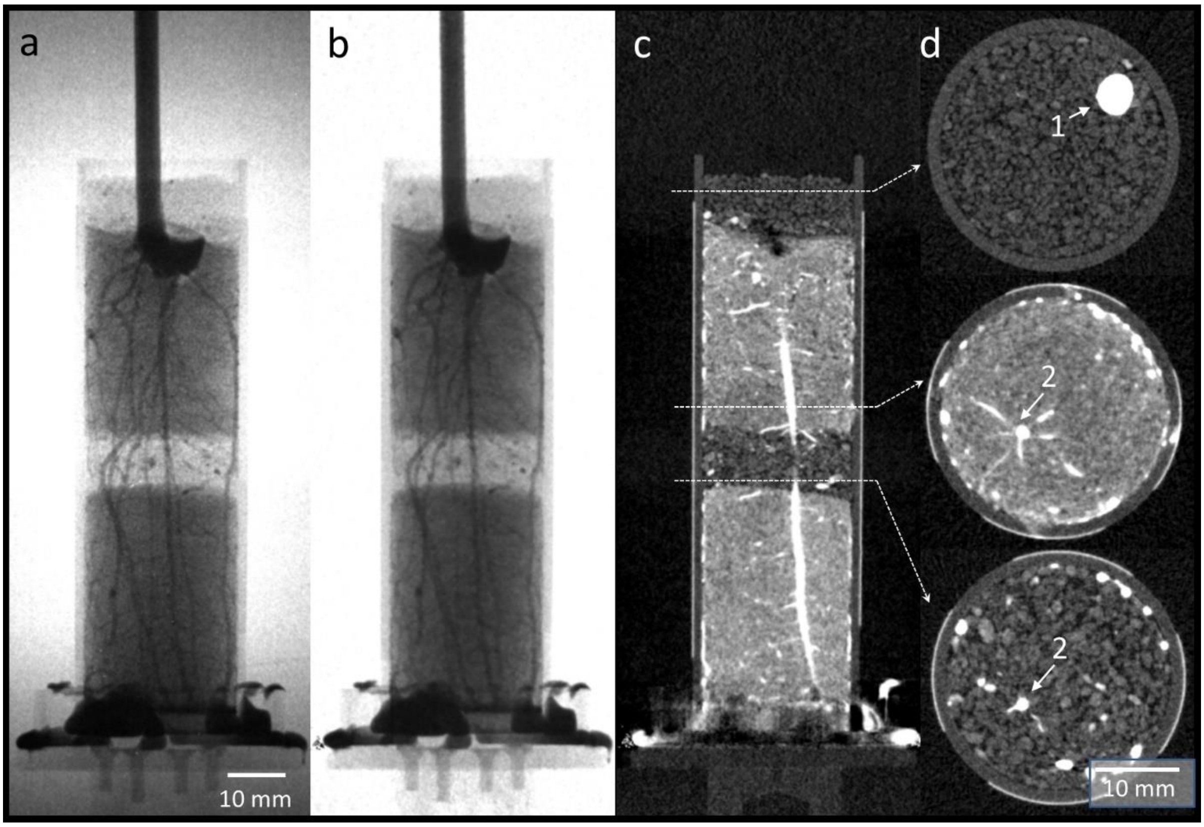

Figure S 3: Illustration of the tomographic acquisition of sample “M14”. (**a**) Raw image (radiographic projection) of a maize plant. (**b**) Normalized radiographic projection; (**c**) Reconstructed vertical slice; (**d**) Reconstructed horizontal slices. Arrows mark the maize stem (1) and the primary root (2).

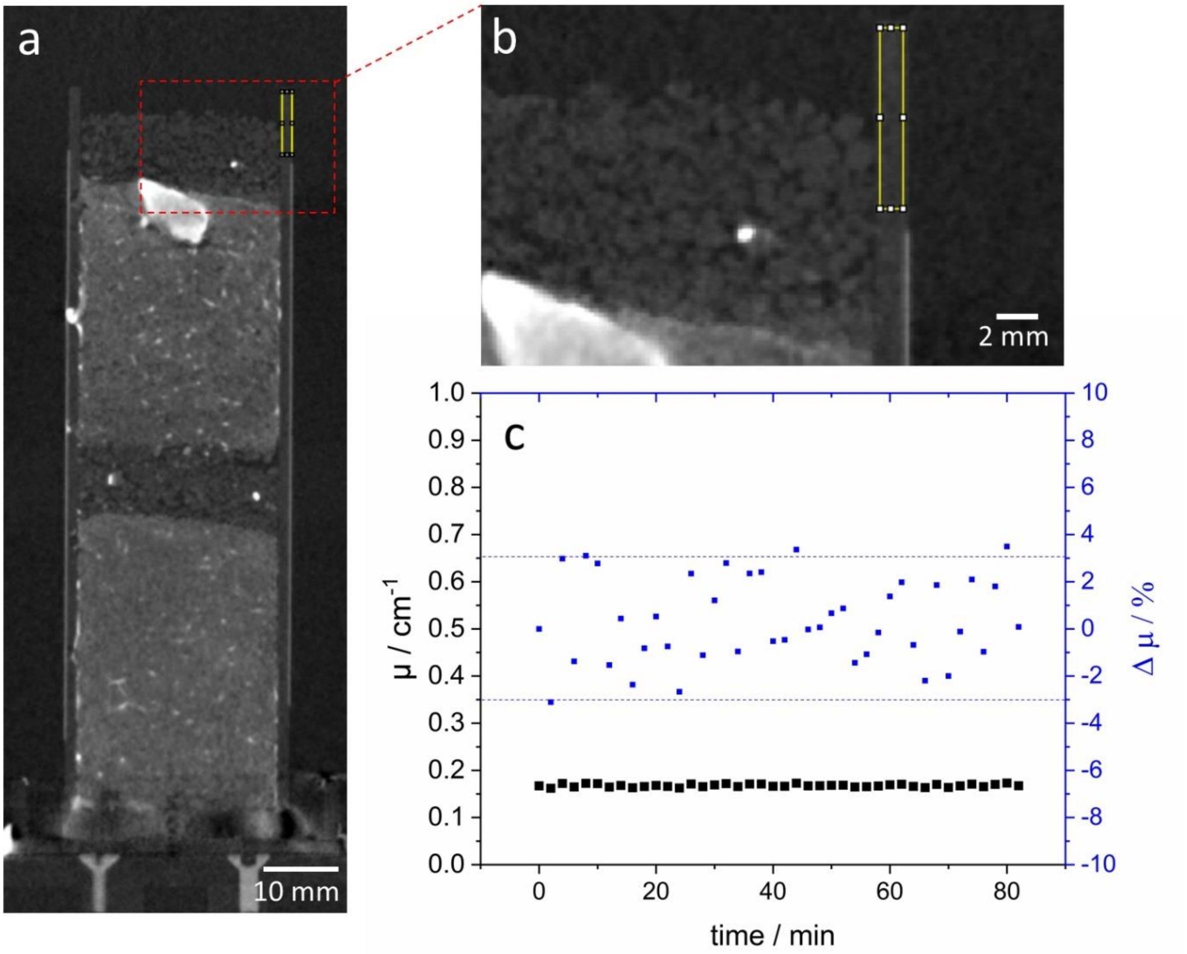

Figure S4: Evaluation of temporal deviations of measured neutron attenuation coefficients using a reference sub-volume of sample "M14". (**a**+**b**) Vertical tomographic slice of cylindrical quartz glass vessel containing the plant sample. A section of the container wall (marked in yellow) represents a reference sub-volume with uniform and invariant material properties. (**c**) Neutron attenuation coefficient and percentage deviation plotted for the yellow-marked section of the course of experiment. The relative error due to temporal fluctuations of detector and source is smaller than 2 % (μ_{AVG}= 0.16779 ± 0.00305 cm^{-1}).

Table S1. Key imaging parameters of the fast neutron tomography experiment

Acquisition time per 180°/360° tomogram [s]	60/120
Exposure per radiographic projection [s]	0.2
Pixel size [µm]	55
Binning mode	2 x 2
Physical spatial resolution	220 µm
Number of projections (180°/360°)	300/600
Scintillator type	^{6}LiZnS:Ag
Scintillator thickness [µm]	200
Collimation rate D/L	167
Camera system	sCMOS Andor Neo
Lens system	Nikon 50mm/1.2

Video Caption

Video S1: 3D visualizing of the infiltration experiment showing root system architecture, water infiltration of soil and subsequent uptake and transport of deuterated water inside the roots.
